# Microbiological Quality of Free-Range Eggs from Nest Boxes and Litter in the Late Production Stage in Southeastern Brazil

**DOI:** 10.3390/ani15172597

**Published:** 2025-09-04

**Authors:** Daniel Rodrigues Dutra, Nívea Maria Gomes Misson Carneiro, Erick Alonso Villegas-Cayllahua, Heloisa de Almeida Fidelis, Érika Nayara Freire Cavalcanti, Romário Alves Rodrigues, Nadir Staidler Bornatte, Marco Antonio de Andrade Belo, Hirasilva Borba

**Affiliations:** 1 Faculty of Agriculture and Veterinary Sciences, São Paulo State University, Jaboticabal 14884-900, Brazil; 2 Independent Researcher, Londrina 86061-280, Brazil

**Keywords:** alternative production, egg microbiology, egg quality, eggshell, non-cage system, *Salmonella*

## Abstract

This study assessed the microbiological quality of free-range eggs produced in Southeastern Brazil, focusing on the effects of collection location. Eggs were collected from either nest boxes (designated laying areas containing clean substrate) or from the bedding substrate (litter-covered floor of the poultry house). Eggs from the bedding had significantly higher counts of thermotolerant coliforms, psychrotrophs, and *Staphylococcus* compared to nest-collected eggs. Across all evaluated microorganisms, eggshells showed greater contamination than the internal contents. Bedding substrates showed higher counts of mesophilic aerobes, psychrotrophs, *Staphylococcus*, and *Clostridium* compared to nest box substrates. Eggs from the bedding showed compromised internal microbiological quality, with coliform values exceeding 2 log10 most probable number (MPN) per mL. Further, 10% of the evaluated pools were suggestive of the presence of *Salmonella* spp., in the internal components of bedding eggs and on the shells of nest eggs. Therefore, it is strongly recommended that eggs be collected from nest boxes right after laying, while eggs laid on the bedding substrate should be excluded from human consumption.

## Simple Summary

This study investigated the microbiological quality of eggs produced in a commercial free-range system in Southeastern Brazil during the late production stage, evaluating the effects of collection location. Eggs were collected either from nest boxes (designated laying areas with a clean substrate) or from the bedding substrate, referring to the litter-covered floor of the barn. The results revealed high microbial counts, especially coliforms, in both nest and bedding substrates, with coliform levels exceeding safe thresholds. Additionally, 10% of the samples tested positive for *Salmonella* spp., either in bedding eggs or on the shells of nest eggs. No contamination from sulfite-reducing *Clostridium* was found. The study concluded that hygienic conditions were insufficient, emphasizing the need for better food safety practices in non-cage systems. It is recommended that free-range eggs be collected immediately after laying to minimize contamination. The consumption of eggs collected directly from bedding is not recommended due to the increased risk of microbial contamination.

## 1. Introduction

Brazil is responsible for producing over 52 billion eggs annually, thanks to the housing of 1.3 million laying hens across the country, making it the fifth largest egg producer in the world [1]. The state of São Paulo alone houses 28.85% of the country’s chicks. However, the consumer market has become increasingly demanding regarding the ethical and sanitary quality of the production systems adopted [2]. The implementation of alternative systems, with a focus on animal welfare, is now a global reality. Leading multinational food companies have committed to sourcing and using only non-cage eggs throughout their supply chains [3].

Moreover, these systems add value to the final product and expand buying and selling options in the egg market. In free-range models, birds can express natural species behaviors, remain loose in barns, have access to grazing areas and perches, and lay their eggs predominantly in nest boxes, and, to a lesser extent, on the aviary bedding, that is, the litter-covered floor of the barn This is typically composed of absorbent material such as rice hulls or wood shavings [4]. On the other hand, eggs produced in these systems are more susceptible to microbial contamination due to their frequent contact with feces and other environmental contaminants, especially during the late production stage [5,6,7].

Contamination arising from inadequate egg handling and the production system itself can lead to numerous foodborne illnesses with various health impacts on consumers, highlighting the importance of producing safe food from a microbiological standpoint [8]. To measure the microbiological quality of animal-derived products, groups of microorganisms can be used as indicators of the hygienic−sanitary conditions of both the environment and the product itself [9]. These microorganisms include mesophiles, psychrotrophs, *Staphylococcus* spp., *Salmonella* spp., *Clostridium* spp., thermotolerant and total coliforms.

Thus, the objective of this study was to describe and evaluate the influence of the laying location on the microbiological quality of eggs produced in a free-range system in Southeastern, Brazil, during the late laying phase.

## 2. Materials and Methods

### 2.1. Location and Period of Experiment

All research was approved by the Animal Ethics Committee (CEUA) of the Faculty of Agricultural and Veterinary Sciences of São Paulo State University (FCAV/UNESP) prior to the start of data collection (protocol no. 1685/21).

The study was conducted on a commercial animal welfare model farm operating under a free-range production system, located approximately 22 km from the municipality of Jaboticabal, São Paulo State, Southeastern Brazil. The flock consisted of approximately 4500 Hisex Brown^®^ laying hens (*Gallus gallus domesticus*) at 82 weeks of age, housed in a single batch. The hens had access to perches inside the barn (~396 m^2^, indoor density of 0.088 m^2^/bird), and to an outdoor pasture area (minimum of 1 m^2^/bird), which allowed them to express natural behaviors such as resting, pecking, wing flapping, dust bathing, walking, and sunbathing. The bedding substrate consisted of rice hulls covering the barn floor from the entry of the flock, where some hens laid eggs outside the designated nesting area. Nest boxes, designated as laying sites, were lined with the same substrate, which was renewed every two weeks. Collections took place in October 2021, when the average temperature and humidity in the region were 23.6 °C and 64.7%, respectively [10].

At the beginning of the day, all eggs present in the barn were manually collected according to the farm’s standard management practices and were intended for production. Subsequently, fresh eggs (*n* = 80) were aseptically collected over five periods using a convenience sampling design. Eggs were randomly selected within each pre-identified nest box and directly from the litter—40 units per group, according to De Reu et al. [11].

### 2.2. Nest Substrate Collection

Nest substrate samples were taken using a sterile cotton swab (five layers, 7.5 cm × 7.5 cm) [12]. The swab was gently pressed onto the substrate to ensure full contact, and the samples were then placed in sterile test tubes containing 10 mL of peptone water and sent to the laboratory for immediate analysis [13]. The nest samples were collected in duplicate.

### 2.3. Bedding Substrate Collection

Bedding material was sampled using a drag swab in duplicate, as outlined by SDA/MAPA Ordinance 126/95 [14]. A collaborator, wearing disposable sterile booties, walked across the entire barn, ensuring coverage of the bedding in non-linear patterns. After collection, the booties were placed in sterilized containers with peptone water, sealed, and sent to the laboratory for immediate analysis.

### 2.4. Microbiological Analysis

The collected eggs and substrates were transported aseptically in sterile containers to the Laboratory of Animal-Origin Food and Water Analysis, Department of Preventive Veterinary Medicine and Animal Reproduction, São Paulo State University, Jaboticabal Campus. In the laboratory, the containers were immediately opened under aseptic conditions. Microbiological evaluations were conducted on the external surface of the eggshells, the internal components of the egg (yolk and albumen), and the substrates from the nest boxes and aviary bedding.

The egg samples consisted of a pool of five eggs, which were combined and analyzed together as a single composite sample [15,16]. This pooling approach follows official microbiological monitoring protocols established by current Brazilian legislation, allowing for efficient detection of microbial contamination.

Each pool was individually immersed in 225 mL of buffered peptone water and kept under agitation for one minute, resulting in the first dilution for shell evaluation. The eggs were then removed from the medium and disinfected by spraying with 70% ethanol. After disinfection, the shells were carefully cracked under aseptic conditions to assess the internal content. The internal content of the eggs was mixed and homogenized. For both the eggshell and internal egg components, five serial tenfold dilutions (from 10^−1^ to 10^−5^) were performed. For the bedding substrate and nest material, a larger series of dilutions—12 serial 10-fold dilutions (from 10^−1^ to 10^−12^)—were carried out to accommodate the higher expected microbial load typically found in the environmental samples. The high microbial counts in these substrates required extensive dilution to obtain quantifiable colony counts within the detection limits of the methods used.

The eggs and substrates were subjected to quantitative microbiological analysis for total and thermotolerant coliforms, mesophiles, psychrotrophs, *Clostridium* sulfite-reducing bacteria, and *Staphylococcus* spp., as well as qualitative analysis for *Salmonella* spp., in accordance with Brazilian regulations for microbiological monitoring of products of an animal origin. According to Normative Instruction SDA N. 62, dated 26 August 2003, issued by the Ministry of Agriculture, Livestock, and Supply (MAPA) [17], official microbiological standards for food safety are based on sampling plans that commonly use five analytical units per lot (*n* = 5) for inspection purposes, particularly in the context of regulatory control. Therefore, the results were compared with the official microbiological standards for raw, intact eggs established in Brazil [18,19].

#### 2.4.1. Mesophiles and Psychrotrophs

Aliquots of 1 mL from the dilutions were gently poured and spread onto disposable Petri dishes containing Plate Count Agar (PCA). After allowing the medium to solidify, the plates were incubated at 36 °C for 48 h to enumerate mesophilic bacteria. For psychrotrophic bacteria, 0.1 mL of the dilutions was inoculated onto solidified PCA plates, which were then refrigerated at approximately 4 °C for seven days before colony counting.

#### 2.4.2. *Staphylococcus* spp.

Here, 1 mL of the dilutions was plated on disposable Petri dishes containing Baird-Parker agar and spread carefully with a loop. The plates were incubated at 36 °C for 48 h, and the colonies were counted and characterized using coagulase and catalase tests, following American Public Health Association guidelines [20].

#### 2.4.3. *Coliforms* spp.

Then, 1 mL of the dilutions was inoculated into Durham tubes with 9 mL of Lauryl Sulfate Sodium broth and incubated at 36 °C for 48 h. Samples showing gas formation in presumptive tests were transferred to EC Broth and kept in a water bath at 45 °C for an additional 24 h to determine the presence of thermotolerant coliforms. Then, the samples were transferred to Brilliant Green Bile Broth and incubated at 35 °C for 24 h to determine the coliforms, followed by gas formation evaluation. Gas production was assessed by observing visible gas bubbles trapped in the inverted Durham tube within the broth, indicating fermentation activity of coliform bacteria.

#### 2.4.4. *Salmonella* spp.

The initial samples were diluted in 1% peptone water and incubated at 36 °C for 18 h. Subsequently, 0.1 mL of this medium was inoculated into Rappaport broth and 1 mL into Selenite Cystine broth, both incubated in a water bath for 24 h. Finally, the media were plated on Brilliant Green Agar and incubated at 36 °C for 24 h. The results were expressed as the presence or absence of characteristic colonies according to current Brazilian legislation [18,19].

#### 2.4.5. *Clostridium* Sulfite-Reducing Bacteria

Then, 1 mL of the dilutions was plated on disposable Petri dishes with Tryptose−Sulfite−Cycloserine Agar. After the medium solidified, an additional layer was added. Plates were placed in anaerobic jars and incubated for 24 h before reading.

### 2.5. Experimental Design

A completely randomized design in a 2 × 2 factorial arrangement was adopted, with the laying location and egg component established as fixed effects. The main effects and their interactions were tested, and the means were compared using the Tukey test (*p* < 0.05) with the GLM procedure of the SAS statistical package (9.1 version, SAS Institute Inc., Cary, NC, USA). Microbial counts from nesting and litter substrates were compared using Student’s *t*-test.

## 3. Results

### 3.1. Laying Location and Egg Components

The microbiological analysis results revealed significant differences between the egg collection locations and egg components (Table 1). An interaction was observed for *Staphylococcus* spp., with eggshells from the bedding showing higher (*p* < 0.05) counts (5.57 log10 CFU/mL) compared to those from the nests (3.58 log10 CFU/mL). In contrast, the internal components (yolk and albumen) exhibited lower (*p* < 0.05) counts overall, with no significant difference (*p* > 0.05) between the eggs collected from the litter and those from the nests. Characteristic colonies were tested for coagulase production and were negative for coagulase-positive *Staphylococcus* spp. When comparing the laying locations, the eggs collected from the bedding exhibited higher (*p* < 0.05) counts for most microbial groups than those from the nests. Thermotolerant coliforms were significantly higher in the bedding eggs (2.16 MPN/mL) compared to the nest eggs (1.37 MPN/mL; *p* < 0.05). Similarly, psychrotrophs (4.56 vs. 3.47 log10 CFU/mL) were elevated in the bedding eggs. Clostridia were not identified in any of the samples. Regarding the egg components, the eggshell consistently showed higher (*p* < 0.05) microbial counts than the internal contents (yolk and albumen) for all of the evaluated groups. The total coliforms reached 2.46 MPN/mL in the eggshell samples compared to 1.04 MPN/mL in the yolk and albumen. Thermotolerant coliforms were also higher in the eggshells (2.39 MPN/mL) than in the internal components (1.40 MPN/mL). Mesophiles (5.01 vs. 3.60 log10 CFU/mL) and psychrotrophs (5.04 vs. 3.01 log10 CFU/mL) followed the same pattern. Qualitative analysis for *Salmonella* spp. suggested its presence on the shell of eggs collected from the nests, representing 5% of the total samples analyzed. Therefore, the evaluated system showed unsatisfactory microbiological conditions.

### 3.2. Nest and Bedding Substrates

Significant differences were observed in the microbial loads between the two evaluated substrates (nest box and bedding), as presented in Table 2. The bedding showed significantly higher counts of *Staphylococcus* spp., mesophilic, and psychrotrophic bacteria compared to the nest box. *Clostridium* spp. was detected exclusively in the bedding, while it was absent in the samples from the nest box (*p* < 0.05). No significant differences were observed between substrates for the total and thermotolerant coliforms, with both substrates presenting similar values. The search for *Salmonella* spp. suggested the presence of characteristic colonies in the bedding content.

## 4. Discussion

The higher contamination observed in the eggs collected from the litter was expected, as the rice hulls used throughout the 82 weeks of production, combined with the accumulated bird droppings, provided ideal conditions for microbial proliferation on the poultry house floor, including high humidity, temperature, and substrate [21]. Contamination was predominantly observed on the eggshells, which showed higher counts for all analyzed microorganisms compared to the internal components. This indicates that contact with the litter and contaminated droppings is the primary route of contamination, and that the high bacterial load on the shell may facilitate translocation into the internal contents, posing potential spoilage and health risks.

This general pattern of contamination is exemplified by the counts of *Staphylococcus* spp. on the eggshells, which were particularly high in the eggs collected from the bedding, whereas the eggshells from nests showed lower values. These findings reflect the elevated microbial loads found in the nests and bedding and are consistent with the observations of Rumão et al. [22]. Internally, counts of *Staphylococcus* spp. were low for both laying locations, indicating limited vertical penetration. Commonly observed in laying houses during warmer seasons, they are mesophilic bacteria capable of producing heat-resistant enterotoxins involved in various cases of food poisoning. The longer the exposure of eggs to warm environments, the greater the risk of contamination by *Staphylococcus* spp. and, consequently, food poisoning cases [23]. Primary contamination of free-range eggs by *Staphylococcus* spp. is facilitated by their presence in the birds’ microbiota, representing a critical point in maintaining food safety [24]. Staphylococci are Gram-positive cocci, facultative anaerobes with a preference for aerobic conditions where they can produce catalase, an important enzyme that aids in differential diagnosis from non-catalase-producing cocci, such as bacteria of the genus *Streptococcus*. In this study, coagulase and catalase tests were negative. Despite this, recent records have shown that coagulase-negative *Staphylococcus* are also involved in outbreaks of foodborne diseases [25,26]. Although Normative Instruction No. 161/2022 does not have legal standards for *Staphylococcus* spp. in the sale of intact raw eggs, values below 4.00 log10 are considered microbiologically safe for food, representing a low risk of staphylococcal poisoning [27]. Higher values were recorded for the eggshells from the aviary bedding, highlighting the importance of selling and consuming eggs preferably collected from nest boxes. Furthermore, the high counts of *Staphylococcus* spp. in the analyzed substrates could be related to the nutrient-rich environment and high bird density, facilitating their proliferation.

The high contamination of bedding and nest by coliforms also highlights unsatisfactory hygienic−sanitary conditions due to high fecal contamination by enteropathogens. Coliforms, belonging to the *Enterobacteriaceae* family, stand out among the microbiological indicators of food quality, indicating the sanitary conditions of the product and the presence of fecal or environmental pathogens capable of causing harmful effects on human health. Normative Instruction No. 161/2022 [19] sets a maximum value for *Enterobacteriaceae* at 2.00 log10/g in intact raw eggs, indicating that the eggs from bedding had unacceptable quality according to current legislation. This indicates that the eggs collected from bedding are highly susceptible to coliform contamination and are therefore not recommended for consumption, representing a high risk to public health.

Generally, it is accepted that a chicken egg is sterile until the time of laying [28]. However, rapid contact of the egg’s outer surface with fecal material accumulated in the aviary bedding was sufficient to contaminate the shell with total and thermotolerant coliforms. Moreover, exposure to the bedding may lead to contamination of the internal contents of the eggs, as these microorganisms can infiltrate through the shell pores over time [29]. Once they pass through the membranes, coliforms multiply in the albumen until they reach the yolk, which is considered an excellent growth medium [30]. This risk is particularly relevant for eggs laid by older hens, which tend to have thinner and more porous shells due to age-related degradation of the shell ultrastructure. In addition, both the coverage and thickness of the cuticle are reduced as hens age, further compromising the eggshell’s barrier function and facilitating microbial penetration [7,31]. This class of pathogens is divided into two major groups according to their resistance to ambient temperature: total coliforms and thermotolerant [32]. Total coliforms can produce gas through lactose fermentation when incubated. Among the genera that compose this group, those of greatest prevalence are *Escherichia*, *Enterobacter*, *Citrobacter*, and *Klebsiella*. Except for the genus *Escherichia*, present in the gastrointestinal tract of birds, the others are found in the soil and nests, hence their relevance in evaluating the hygienic−sanitary conditions of non-cage alternative systems. Unlike total coliforms, thermotolerant coliforms can produce gas through lactose fermentation. Most of this group belongs to the genus *Escherichia*, mainly *E. coli*, considered a crucial indicator in the occurrence of fecal contamination in fresh animal-origin foods, such as eggs, reflecting the potential risks of the proliferation of pathogenic and toxigenic microorganisms in the final product [33]. When combined with the total coliform count, they provide a general overview of the microbiological quality of the food offered to consumers. In this context, it is possible to affirm that the high load of total and thermotolerant coliforms in the nest and bedding implies problems in the microbiological quality of the eggs, which was to be expected in a system where the birds roam and excrete freely throughout the barn.

The higher bacterial load of mesophiles and psychrotrophs on eggs from the litter can be also attributed to direct contact with fecal matter and environmental contaminants, as observed by Jones et al. [34] and Campbell et al. [5]. For psychrotrophs, internal counts were similar for both nest and bedding eggs, indicating that high environmental microbial loads facilitate their translocation, even at room temperature. This may be influenced by bird age, as older hens produce eggs with thinner shells or altered cuticle composition, potentially increasing contamination [7]. The elevated mesophile and psychrotroph levels in bedding and nest substrates further confirm the high microbial load in the system.

Aerobic mesophilic counts serve as general hygiene and quality indicators because they proliferate at non-refrigerated temperatures, increasing with prolonged environmental exposure and room temperature storage, while refrigeration suppresses growth [35,36]. In Brazil, eggs are marketed without refrigeration, making assessment of these groups crucial for consumer health. Psychrotrophics, including *Pseudomonas*, *Micrococcus*, and *Flavobacterium*, multiply under refrigeration, contributing to the spoilage of refrigerated eggs [34,37] and posing risks if eggs are consumed without adequate cooking [38]. Although there are no legal standards for mesophiles and psychrotrophs in Brazil, the counts were higher than those reported by Lima et al. [39] in Recôncavo da Bahia, Brazil (1.81 and 1.56 log10 CFU/g, respectively) and similar to counts found by Melo et al. [40] in Seropédica, Brazil. These findings confirm that mesophilic and psychrotrophic bacteria are key indicators of free-range egg microbiological quality and shelf life [37].

The results also suggested the presence of *Salmonella* spp. in 10% of the evaluated pools, corresponding to eggshells collected from nest and internal components of eggs from the bedding, indicating possible vertical contamination and even contamination of the bedding material. Egg contamination can occur even before oviposition when the reproductive tract is colonized by *Salmonella* spp. [41]. Several authors have reported the contamination of free-range eggs by *Salmonella* spp. [34,42]. Similar results were observed by Parisi et al. [42], who detected its presence in 2.36% of free-range eggs. Degenhardt and Pereira [43] also identified the presence of *Salmonella* spp. on the shells and internal components of commercialized free-range eggs. Complementary tests indicated that the species found in the samples had characteristics similar to *Salmonella* spp. from wild and domestic birds, which may not necessarily pose a threat to consumer health. Since the absence of *Salmonella* spp. is the only microbiological standard required for the commercialization of whole raw eggs in Brazil [18,19], the evaluated alternative system did not meet the legal requirements for food safety and, therefore, presents unsatisfactory microbiological conditions [34,43].

The genus *Salmonella* spp., belonging to the *Enterobacteriaceae* family, consists of Gram-negative bacilli that have the gastrointestinal tract of humans and animals as their main reservoir. Its significance for public health is such that the absence of this bacterium is essential for the commercialization of whole raw eggs in Brazil, as established by RDC 724/2022 and IN 161/2022 of the National Health Surveillance Agency [18,19]. The main consequences of salmonellosis include typhoid fever, enteric fever, and enterocolitis [33]. The bacteria penetrate the cells of the digestive tract, where they multiply and trigger an inflammatory process that stimulates diarrhea through hypersecretion [44]. Depending on the *Salmonella* spp. serotype ingested, this condition can develop into severe and even fatal cases. Therefore, analyzing the microbiological quality of eggs in non-cage production systems is crucial to ensuring consumer safety [45]. It is important to highlight the need for more precise methodologies to identify the species of *Salmonella* spp. and its serovars, and to determine their pathogenic potential to humans, an aspect not covered by current legislation. Moreover, the suggestive presence of *Salmonella* spp. in the nest underscores the importance of adopting appropriate sanitary practices and acquiring quality materials used to line the nesting boxes. These measures are essential to prevent future contamination and ensure the safety of products available on the market.

Regarding the *Clostridium* genus, the continuous contact of laying hens with the soil predisposes the birds to exposure to this bacteria group, responsible for more than 2 billion dollars in poultry industry losses annually [46]. The primary habitat of this genus is the soil, which presents a challenge for free-range systems, in which birds have access to the ground and pasture areas [33]. Clostridia are microorganisms capable of spore production, which gives them great resistance to adverse environmental conditions. However, the found results were satisfactory, as none of the eggs collected from bedding or nest boxes showed colony-forming units for sulfite-reducing *Clostridium*, corroborating the findings of Rossi and Bampi [47] when analyzing eggs from Santa Catarina, southeastern Brazil. Additionally, contamination by *Clostridium* can also occur indirectly due to the presence of soil particles on the exterior of the food. Comprised mostly of Gram-positive bacilli and anaerobic, the genus includes two main pathogenic species, *C. botulinum* and *C. perfringens*, among others with a high spoilage capability. *C. botulinum* toxins can cause botulism, a disease of extreme public health interest due to the severity of its symptoms and high mortality rate. *C. perfringens* can cause two types of foodborne illnesses: classic intoxication, characterized by mild gastrointestinal disorder, and necrotic enteritis, which is severe. Birds may not show noticeable symptoms, yet still shed the bacteria in their excreta [48], which, when in contact with nesting and bedding material, could contaminate the eggs themselves [33]. Therefore, these findings provide an encouraging perspective regarding the absence of this pathogen in eggs produced under free-range systems.

The importance of using quality bedding materials and continuously monitoring the hygienic conditions of the eggs, equipment, and facilities, particularly the floor and nest boxes, is emphasized to reduce microbial load in free-range systems and ensure product safety. In commercial laying hen farms, common bedding materials include wood shavings, rice hulls, straw, sand, or sawdust, depending on the region and farm management practices. In this study, rice hulls were used as the bedding substrate, which may be a source of contamination, as rice fields attract wild birds and rodents, vectors of many pathogens [49].

Although this study assessed free-range eggs from a single commercial farm in Southeastern Brazil, its findings indicate potential health risks relevant to non-cage production systems more broadly. These results underscore the importance of stringent sanitary monitoring of eggs, nest boxes and litter in free range systems, where the focus on animal welfare may inadvertently increase contamination risks. Implementing proper egg sanitization before consumption is recommended to reduce bacterial infection and foodborne illness. While this study did not evaluate specific disinfection methods, future research should address their effectiveness and producers should adopt good management practices to limit environmental exposure of eggs after laying.

## 5. Conclusions

Laying location influences the microbiological quality of free-range eggs during the late production stage, with bedding more contaminated than nest boxes and higher microbial loads on eggshells than in internal components. Nest boxes can also be susceptible to *Salmonella* spp. contamination. Therefore, immediate collection and sanitization are essential to minimize microbial translocation and ensure egg safety, while the consumption of free-range eggs from bedding is not recommended due to the sanitary risk.

## Figures and Tables

**Table 1:** Interaction between laying location and egg component for *Staphylococcus* spp. and means for microbiological count (expressed in log10) in different components of eggs collected from two laying locations. *p*-values are presented.

Egg Component	Laying Location (LL)		*p*-Value				CV (%)
	Nest	Bedding					
	*Staphylococcus* spp. (CFU/mL)		Total	*p* (LL)	*p* (EC)	*p* (LL × EC)	
Eggshell	3.58 ^Ab^	5.57 ^Aa^	4.56	0.000	<0.001	0.002	1.61
Yolk and Albumen	2.00 ^Ba^	2.16 ^Ba^	2.08				
Total	2.79	3.86					
	**Total Coliforms** **(MPN/mL)**	**Thermotolerant Coliforms (MPN/mL)**	**Mesophiles** **(CFU/mL)**			**Psychrotrophs (CFU/mL)**	
Laying location (LL)							
Nest	1.44	1.37 ^B^	3.65			3.47 ^B^	
Bedding	2.06	2.16 ^A^	4.95			4.56 ^A^	
Total	1.75	1.76	4.30			4.02	
Egg component (EC)							
Eggshell	2.46 ^A^	2.39 ^A^	5.01 ^A^			5.04 ^A^	
Yolk and Albumen	1.04 ^B^	1.40 ^B^	3.60 ^B^			3.01 ^B^	
Total	1.75	1.89	4.30			4.02	
*p*-value							
*p* (LL)	0.131	0.052	0.061			0.040	
*p* (EC)	0.002	0.004	0.044			0.001	
*p* (LL × EC)	0.224	0.179	0.804			0.582	
CV (%)	5.04	4.79	3.35			2.71	

Means followed by different letters in columns (uppercase A,B) and rows (lowercase a,b) differ significantly according to Tukey’s test (*p* < 0.05). MPN = most probable number; CFU = colony-forming units. CV = coefficient of variation.

**Table 2:** Microorganism counts (expressed in log10) analyzed in the nest box and bedding substrates.

Analyzed Microorganisms	Substrate		
	Nest Box	Bedding	Total
*Staphylococcus* spp. (CFU/mL)	7.23 ^b^	8.56 ^a^	7.89
Total coliforms (MPN/mL)	12.04	11.66	11.85
Thermotolerant coliforms (MPN/mL)	12.04	11.66	11.85
Mesophiles (CFU/mL)	8.28 ^b^	8.78 ^a^	8.53
Psychrotrophs (CFU/mL)	7.87 ^b^	8.46 ^a^	8.16
*Clostridium* spp. (MPN/mL)	0.00 ^b^	3.53 ^a^	1.76
*Salmonella* spp. (25 g)	Presence	Absence	

^a,b^ Means followed by different letters in the same row are significantly different according to Student’s *t*-test (*p* < 0.05). MPN = most probable number; CFU = colony-forming units.

## Data Availability

The data presented in this study are available upon request from the corresponding author because the data are restricted.
